# Source Tracking and Functional Prediction of Microbial Communities in Initial Jiupei of Fen-Flavor Baijiu: Daqu-Dominated Sourcing with Measurable Environmental Inputs

**DOI:** 10.3390/microorganisms14102306

**Published:** 2026-10-10

**Authors:** Enxiang Zong, Jiaojiao Zhang, Baoyue Zhu, Baoqing Bai, Jinhua Zhang, Ying Zhang, Yukun Yang, Sanhong Fan, Tao Bo

**Affiliations:** 1 School of Food and Health, Beijing Technology and Business University, Beijing 100048, China; zongenxiang@st.btbu.edu.cn (E.Z.); 15393116729@163.com (B.Z.); 2 School of Life Science, Shanxi University, Taiyuan 030006, China; 2024260006@xyafu.edu.cn (J.Z.); baoqingbai@sxu.edu.cn (B.B.); ever840805@sxu.edu.cn (J.Z.); zhangy@sxu.edu.cn (Y.Z.); yangyukun@sxu.edu.cn (Y.Y.); 3 School of Pharmacy, Xinyang Agricultural and Forestry University, Xinyang 464000, China; 4 Xinghuacun College of Shanxi University (Shanxi Institute of Brewing Technology and Industry), Taiyuan 030006, China

**Keywords:** community assembly, environmental microbiota, solid-state fermentation, SourceTracker, starter culture

## Abstract

Understanding the microbial origin of initial fermentation grains (Jiupei) is important for improving process consistency in Fen-flavor Baijiu, yet the relative contributions of Daqu and the production environment remain insufficiently resolved. In this study, high-throughput sequencing, community ordination, SourceTracker analysis, co-occurrence network analysis, and PICRUSt2-based functional prediction were used to characterize the bacterial and fungal communities in initial Jiupei (JP0d), Daqu, and representative environmental reservoirs. Daqu was inferred as the dominant source, contributing 87.1% of the bacterial and 90.1% of the fungal communities. Environmental reservoirs provided smaller but detectable inputs: fermentation-room air (FJSKQ) contributed 2.53% of the bacterial community, equipment surfaces accounted for 1.1–5.1% of the bacterial community, and tools (GJ), cooling conveyors (LSJ), and distillation surfaces (ZT) accounted for 1.5–3.2% of the fungal community. The JP0d–JP28d correlation was higher for bacteria (r = 0.46) than for fungi (r = 0.39), indicating greater end-point compositional similarity for bacteria in this dataset. Functional prediction identified prominent pathways related to amino acid and carbohydrate metabolism, membrane transport, translation, replication, and repair in the JP0d bacterial community. These results support a Daqu-dominated source model for the initial Jiupei microbiota and indicate that the production environment may serve as a supplementary microbial reservoir in Fen-flavor Baijiu fermentation. The findings provide a microbial traceability framework for managing open solid-state fermentation systems.

## 1. Introduction

Baijiu is a Chinese distilled spirit produced by solid-state fermentation of cereal substrates, during which mixed microbial communities contribute to ethanol and flavor formation [1,2,3,4]. Fen-flavor (light-aroma) Baijiu uses Daqu as the starter and is fermented in ceramic vats [5,6,7]. Daqu supplies microorganisms and enzymes for saccharification and fermentation, and its composition can vary with starter preparation and type [5,8,9]. Microbial interactions can also alter the metabolism of Baijiu fermentation communities [10,11,12]. Because grain cooling, mixing, transfer, and vat loading expose the substrate to the production environment, the community present in initial fermented grains (Jiupei; JP0d) may reflect inputs from both Daqu and production interfaces [13,14,15]. Identifying these potential inputs can guide where microbiological monitoring is focused, although community profiles alone cannot establish effects on product quality.

Previous work provides important, but differently focused, evidence. Pang et al. (2018) examined environmental microbiota, fermentation communities, and flavor across production periods of light-flavor Baijiu [7]. Studies of Fen-flavor fermented grains have described temporal microbial changes and functional profiles [16], while a recent comparison of Daqu from several regions characterized differences in starter communities and metabolic traits [17]. In other Baijiu systems, Gong et al. (2025) traced environmental contributions to Maotai-flavor Daqu [18]. Cheng et al. (2024) investigated microbial sources and succession in compound-flavor fermented grains [19]. Tang et al. examined microbial sources before and after cellar entry in Furou-type Baijiu [20]. Bo et al. (2024) developed a PMA-based method to characterize living microbial communities in fermented grains [21]. These studies establish the relevance of both starter and environmental microbiota, while differing in aroma type, fermentation stage, sampled source set, or principal analytical question. However, these studies have not quantitatively resolved the relative contributions of Daqu versus environmental sources to the initial fermentation stage of Fen-flavor Baijiu, particularly with respect to the distinct assembly patterns of bacterial and fungal communities.

This study focused on JP0d from a normal Fen-flavor Baijiu fermentation process and compared its bacterial and fungal communities with those of Daqu and representative environmental samples from the production line. High-throughput sequencing was used to characterize microbial diversity and community composition. SourceTracker was then applied to estimate the proportional contributions of potential sources to JP0d. Correlation analysis between JP0d and later fermentation stages was used to assess the degree of compositional similarity of bacterial and fungal communities across time points. Finally, co-occurrence network analysis and PICRUSt2-based functional prediction were used to explore potential microbial associations and functional profiles in JP0d. While source-tracking approaches have been applied to other Baijiu flavor types, the microbial source structure of initial Jiupei in Fen-flavor Baijiu has not been quantitatively resolved. Specifically, the relative contributions of Daqu and environmental reservoirs to JP0d, and whether these contributions differ between bacterial and fungal communities, remain unknown. This study addresses this gap by providing the first quantitative source-tracking analysis of initial Jiupei in Fen-flavor Baijiu. Clarifying the microbial source structure of initial Jiupei in Fen-flavor Baijiu is therefore important for improving fermentation consistency and product quality in industrial production.

## 2. Materials and Methods

### 2.1. Experimental Design and Sample Collection

Sampling was conducted at the Xinghua Village Fenjiu Factory in Shanxi Province. Initial Jiupei samples (JP0d) were collected on day 0 of a routine Fen-flavor Baijiu fermentation during the Yuanpai phase (the production period between the Lipai period, which begins in September after the summer production break, and the Tiaopai period, which is the last period before the summer break [7]; this phase is also characterized by relatively stable production conditions). For the temporal succession analysis, Jiupei samples were additionally collected at multiple time points during the fermentation course: day 3 (JP3d), day 7 (JP7d), day 11 (JP11d), day 15 (JP15d), day 21 (JP21d), day 24 (JP24d), and day 28 (JP28d). At each time point, approximately 100 g of Jiupei was aseptically sampled from the center of the fermentation vat at a depth of 15–20 cm below the surface.

**Figure 1:**
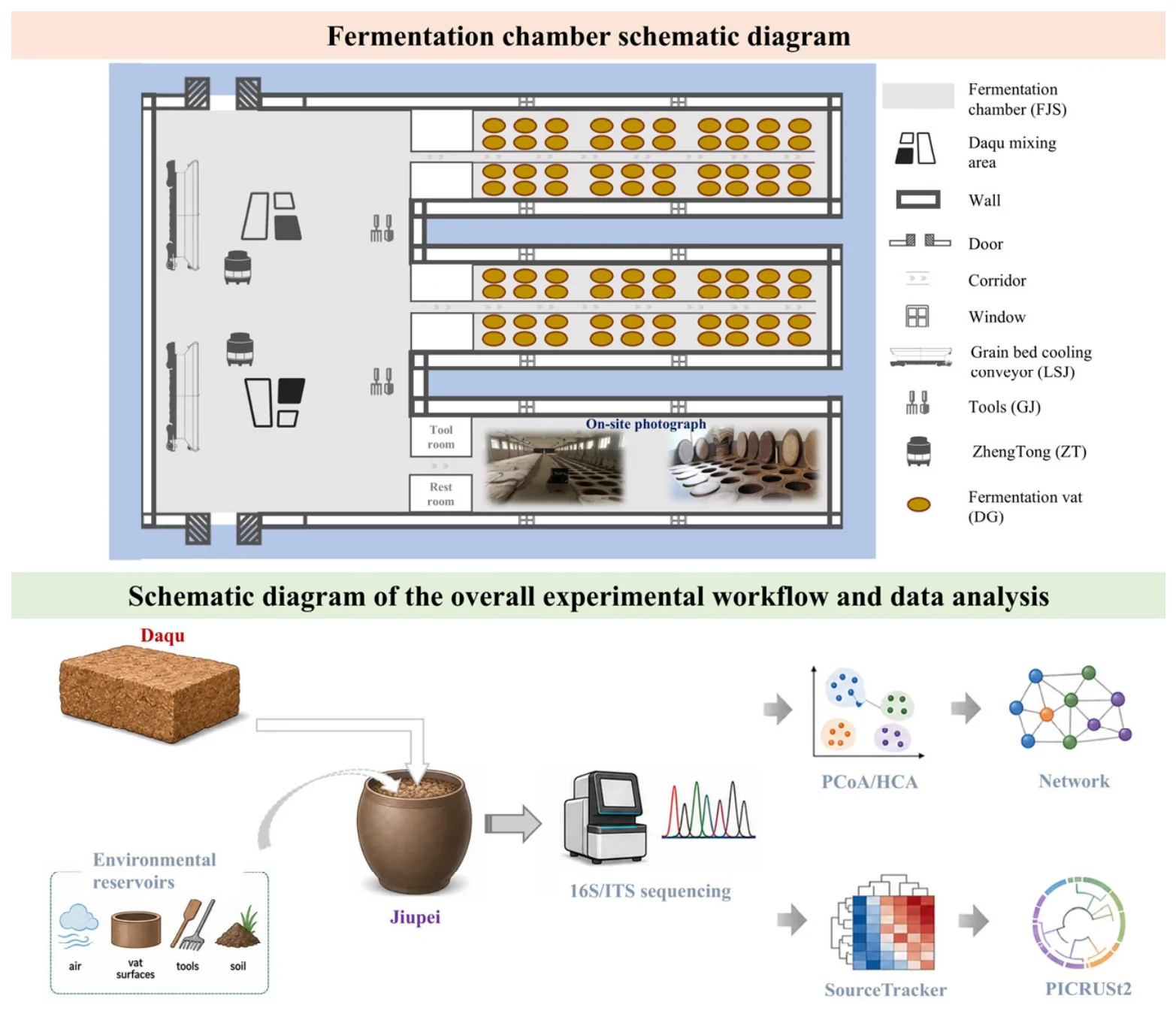
Schematic diagram of the experimental design and overall workflow.

From the same batch, 100 g of the corresponding Daqu (DQ) was sampled. Environmental samples were collected immediately before fermentation initiation. Cotton swabs pre-moistened with 0.1 M phosphate-buffered saline (PBS) were used to sample the following surfaces: production tools (GJ), cotton quilts (MB), the grain bed cooling conveyor (LSJ), ZhengTong (ZT; a traditional batch steamer–distiller used in Baijiu production), the floor (DM), the edge of the fermentation vat (DGBY), and the inner wall of the fermentation vat (DGNB). Adjacent soil (TR) was also sampled. All Jiupei samples were collected from the same fermentation chamber, where multiple parallel fermentation vats were operated under identical conditions. At each time point, three independent fermentation vats were sampled as biological replicates. Airborne microbes were sampled from the fermentation chamber (FJSKQ) onto gelatin filters using a Sartorius MD8 air sampler (Sartorius AG, Göttingen, Germany) operating at a flow rate of 50 L/min for 40 min. The FJSKQ air filter did not yield a measurable fungal sample for ITS community profiling; accordingly, FJSKQ was excluded from fungal analyses and fungal SourceTracker candidate sources. All samples were aseptically packaged, transported on ice, and stored at −80 °C until DNA extraction. Three technical replicates were collected at each time point for sequencing (Figure 1).

### 2.2. Genomic DNA Extraction and High-Throughput Sequencing

Genomic DNA was isolated from all samples using the FastDNA SPIN Kit for Soil (MP Biomedicals, Santa Ana, CA, USA) according to the manufacturer’s protocol. DNA concentration and purity were measured using a NanoBio 200 spectrophotometer (OPTOSKY, Xiamen, China), and DNA integrity was assessed by 1% agarose gel electrophoresis. Qualified DNA samples were stored at −80 °C before downstream analysis. For bacterial community analysis, the V4 region of the 16S rRNA gene was amplified using primers 515-F (5′-GTGCCAGCMGCCGCGGTAA-3′) and 806-R (5′-GGACTACHVGGGTWTCTAAT-3′). For fungal community analysis, the ITS region was amplified using ITS-1F (5′-CTTGGTCATTTAGAGGAAGTAA-3′) and ITS-2R (5′-GCTGGTTCTTCATCGATGC-3′). PCR amplification followed established laboratory protocols [22]. Each 30 μL PCR reaction contained 15 μL of Phusion High-Fidelity PCR Master Mix (New England Biolabs, Beijing, China), 0.2 μM of each primer, and 10 ng of template DNA. The thermal program included initial denaturation at 98 °C for 1 min, followed by 30 cycles of 98 °C for 10 s, 50 °C for 30 s, and 72 °C for 30 s, with a final extension at 72 °C for 5 min. Sequencing libraries were constructed using the NEBNext Ultra DNA Library Prep Kit for Illumina (NEB, Ipswich, MA, USA). Library quality was assessed using an Agilent 2100 Bioanalyzer (Agilent Technologies, Inc., Santa Clara, CA, USA), and library concentrations were quantified by real-time PCR. Qualified libraries were sequenced on an Illumina HiSeq 4000 platform (Novogene, Tianjin, China).

### 2.3. Sequence Quality Control and Bioinformatic Analysis

Raw sequencing data were processed by removing primer and barcode sequences. Reads shorter than 40 bp or containing at least 8% ambiguous bases were discarded using Readfq (V38, https://github.com/cjfields/readfq (accessed on 5 January 2024)). Chimeric sequences were then removed to obtain high-quality clean reads. Amplicon sequence variants (ASVs) were generated from clean reads and used for downstream community analysis. Taxonomic classification was performed using the classify-sklearn algorithm in QIIME2 (version 2024.2) [23,24]. Bacterial ASVs were assigned using the SILVA database (version 138.2), and fungal ASVs were assigned using the UNITE database (version 9.0).

### 2.4. Statistical Analysis and Visualization

Observed ASVs and Shannon indices were calculated in QIIME2 to compare microbial richness and diversity among sampling sites. Weighted UniFrac distances were calculated for both bacterial and fungal communities, followed by principal coordinates analysis (PCoA) and hierarchical clustering analysis (HCA) in R (version 4.3.1). PCoA visualization was performed using the ade4 (version 1.7‑22) and ggplot2 (version 3.4.4) packages. Correlation heatmaps were generated using the pheatmap (version 1.0.12) package in R. Pairwise Spearman correlations among JP0d, Daqu, environmental samples, and subsequent Jiupei samples were calculated to evaluate community similarity. SourceTracker (version 0.9.8) was used to infer microbial source contributions to JP0d, with JP0d designated as the sink and Daqu and environmental samples designated as sources.

For network construction, bacterial and fungal ASVs were aggregated at the genus level to reduce data sparsity and minimize spurious correlations arising from zero-inflated ASV-level counts. Genera with mean relative abundance below 0.1% or detected in fewer than 30% of JP0d samples were excluded. Spearman’s rank correlation was applied to the filtered genus-level data, and correlation pairs with |r| > 0.6 and FDR-adjusted q < 0.05 were retained as edges. Networks were visualized using Gephi (version 0.9.2), and topological metrics were calculated to describe network properties.

Functional potential was predicted using PICRUSt2 (version 2.5.0) and mapped to Kyoto Encyclopedia of Genes and Genomes (KEGG) pathways. These predicted functions were interpreted as potential functional profiles rather than direct measurements of gene expression or metabolic activity.

## 3. Results and Discussion

### 3.1. Microbial Alpha Diversity Differed Among Daqu, Initial Jiupei, and Environmental Reservoirs

**Figure 2:**
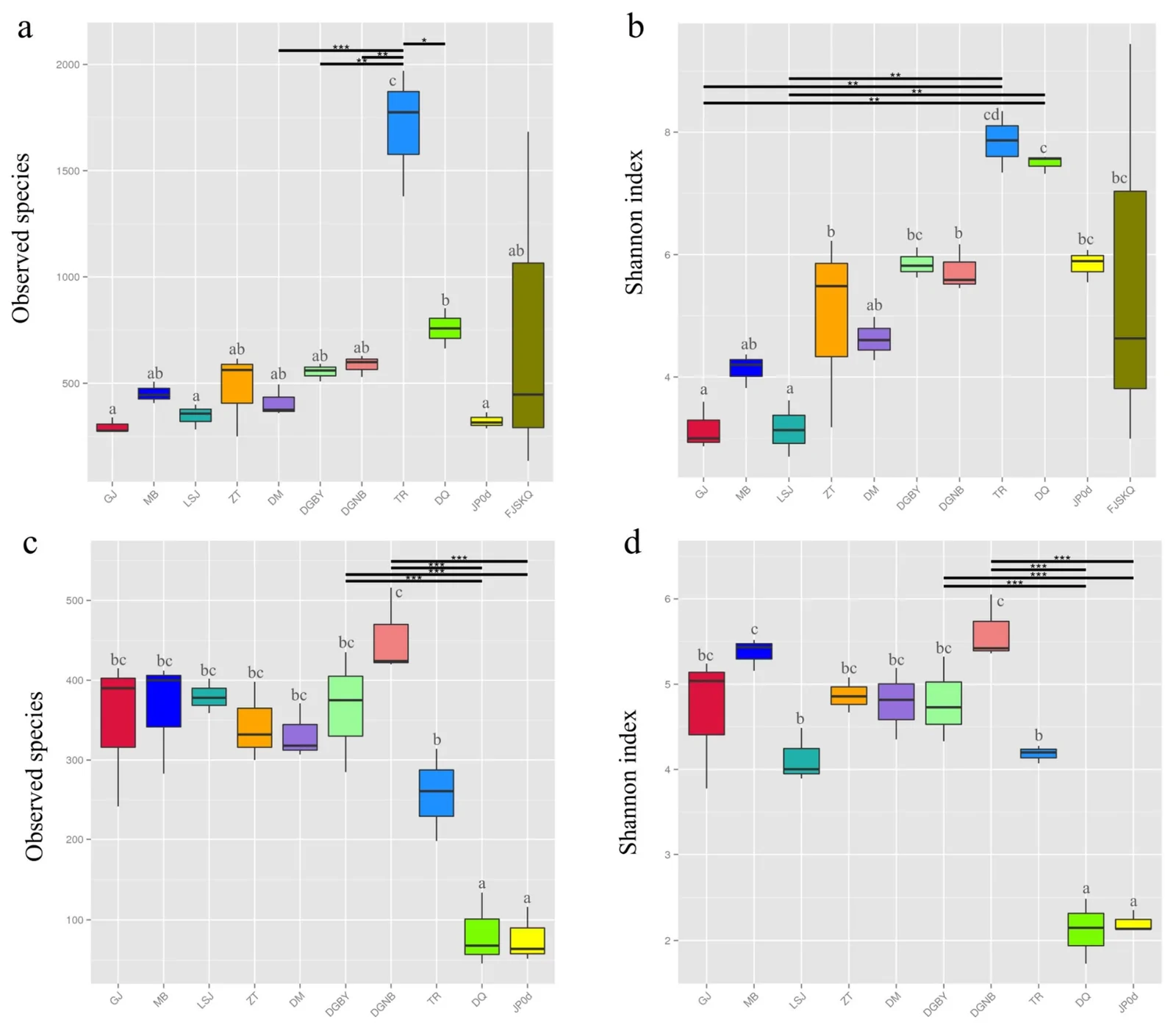
Alpha-diversity analysis of microbial communities. (**a**) Observed bacterial ASVs; (**b**) bacterial Shannon index; (**c**) observed fungal ASVs; (**d**) fungal Shannon index. Different letters above sample groups indicate significant differences (*p* < 0.05), as determined by one-way ANOVA followed by Tukey’s post hoc test. Asterisks indicate significant differences between specific pairs: * *p* < 0.05, ** *p* < 0.01, *** *p* < 0.001.

Rarefaction curves for both bacterial and fungal communities reached saturation (Figure S1), indicating that the sequencing depth was sufficient for diversity analysis. The TR group showed the highest bacterial richness, with a mean of 1707 observed ASVs, which was significantly higher than that of the other groups (*p* < 0.01). The GJ group had the lowest bacterial richness (292 ASVs), whereas DQ and JP0d contained 757 and 320 ASVs, respectively. The difference between DQ and JP0d was significant (*p* < 0.05) (Figure 2a; Table S1). Fungal richness was lowest in DQ (82 ASVs) and JP0d (77 ASVs), and both were significantly lower than the environmental groups (*p* < 0.05) (Figure 2c; Table S1). The Shannon index was highest in TR for bacteria, whereas for fungi, the highest Shannon indices were observed in DGNB and MB. DQ and JP0d did not differ significantly in Shannon diversity, but their fungal diversity was lower than that of the environmental reservoirs (Figure 2b,d). These results indicate that environmental reservoirs, especially soil, contained more diverse microbial communities, whereas Daqu and JP0d harbored communities with lower richness at the start of fermentation.

### 3.2. Bacterial Communities Showed Similarity Between Daqu and Initial Jiupei and Convergence Across Production Surfaces

**Figure 3:**
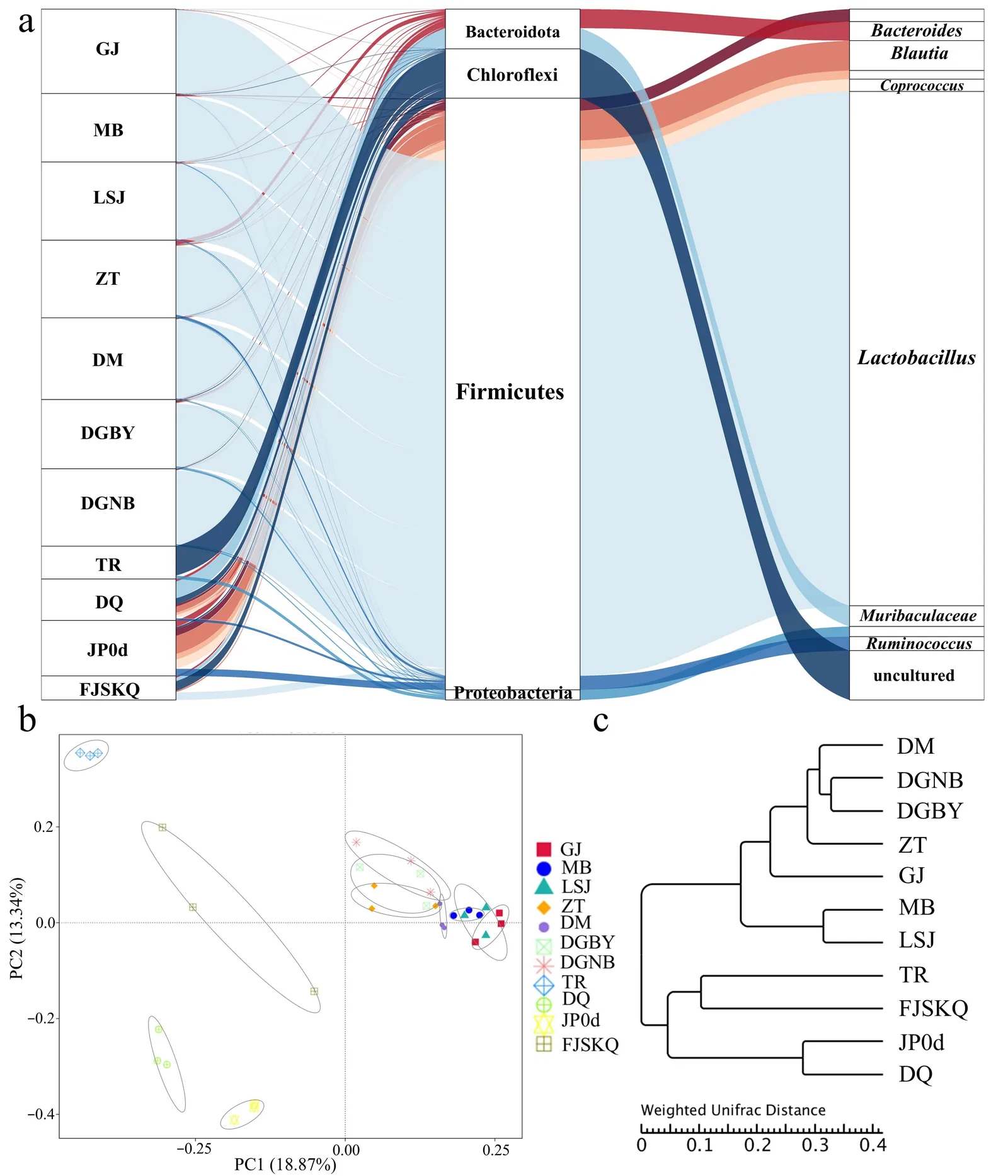
Bacterial community composition of JP0d, Daqu, and environmental samples. (**a**) Sankey diagram showing taxonomic flow. (**b**) PCoA based on weighted UniFrac distances. (**c**) Hierarchical clustering analysis (HCA).

Across JP0d, DQ, and environmental samples, the dominant bacterial phyla were Firmicutes, Proteobacteria, and Bacteroidota. Firmicutes dominated most samples, exceeding 50% relative abundance in GJ, ZT, DM, DGBY, DQ, and JP0d. In contrast, Proteobacteria dominated TR (43.3%), whereas Firmicutes accounted for only 4.30% in this group. Chloroflexi was enriched in TR and was not detected in GJ or JP0d. Bacteroidota showed relatively high proportions in DQ (18.0%) and JP0d (7.1%) (Figure S2a). At the genus level, *Lactobacillus* was abundant on several production surfaces, including GJ, MB, LSJ, ZT, DM, DGBY, and DGNB, where it accounted for more than 70% of the community (Figure 3a). In contrast, *Lactobacillus* was less abundant in DQ and JP0d. This pattern is consistent with previous observations that *Lactobacillus* is present at low abundance in mature low-temperature Daqu for light-flavor Baijiu [5]. Because *Lactobacillus* often increases during Baijiu fermentation, its enrichment on equipment and surface samples may indicate that production interfaces can retain or redistribute fermentation-associated bacteria across brewing cycles.

PCoA based on weighted UniFrac distances showed tight clustering among replicates from the same sampling point, indicating good intra-group reproducibility (Figure 3b). DQ and JP0d formed distinct but closely positioned clusters, suggesting similar bacterial community structures. TR and FJSKQ were clearly separated from other samples, indicating distinct bacterial profiles. HCA further grouped the samples into three major branches: JP0d with DQ, FJSKQ with TR, and the remaining environmental samples (Figure 3c). Within the environmental branch, LSJ and MB clustered together, whereas DGNB, DGBY, and DM formed a closely related subgroup. This spatial pattern suggests that bacterial communities on adjacent or functionally connected production interfaces may converge through repeated contact, dispersal, and surface colonization. The similarity between DQ and JP0d supports the role of Daqu as the main initial inoculum, while the clustering of vat-associated samples indicates that the production environment may provide supplementary microbial inputs in the open fermentation system.

### 3.3. Fungal Communities Were Dominated by Ascomycota and Showed Reservoir-Specific Patterns

**Figure 4:**
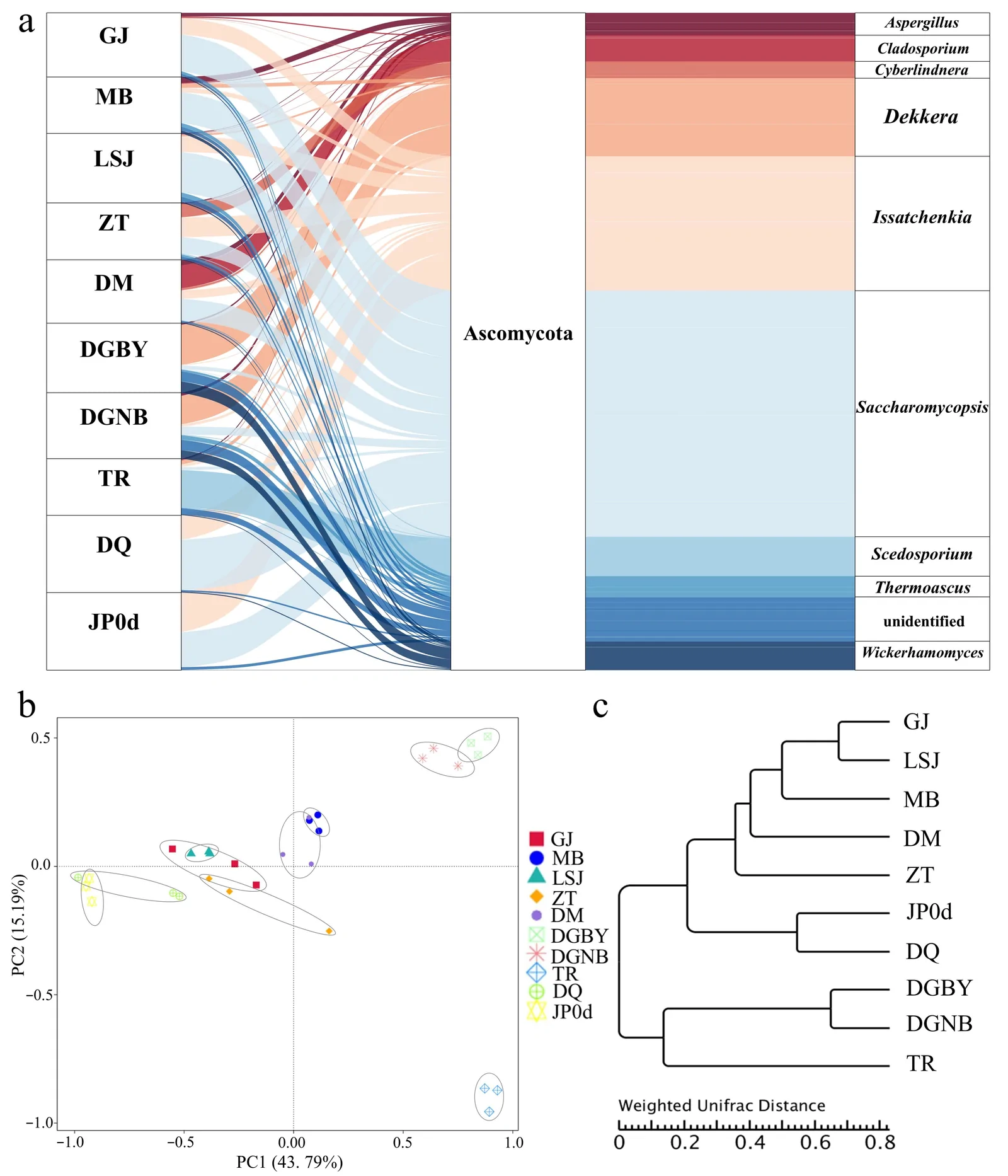
Fungal community composition of JP0d, Daqu, and environmental samples. (**a**) Sankey diagram showing taxonomic flow. (**b**) PCoA based on weighted UniFrac distances. (**c**) Hierarchical clustering analysis (HCA).

Ascomycota dominated the fungal communities in all samples, accounting for more than 80% of the relative abundance and reaching 97.6% in DQ. Basidiomycota was the second most abundant phylum in environmental samples, accounting for 3.7–13.0%, whereas Mucoromycota was detected at appreciable levels in JP0d, DQ, LSJ, ZT, and GJ (Figure S2b). At the genus level, *Saccharomycopsis* and *Issatchenkia* were predominant in JP0d and DQ, together accounting for more than 75% of the fungal community (Figure 4a). Both genera were also detected in environmental samples at lower abundance. Cladosporium was enriched in DM (28.8%), whereas *Wickerhamomyces* reached 14.6% and 10.8% in DGBY and DGNB, respectively. In JP0d, *Wickerhamomyces* remained low (0.3%). *Saccharomycopsis* and *Issatchenkia* are important functional fungi in Baijiu production and are associated with starch degradation and ethanol production [16,25,26]. The low abundance of *Wickerhamomyces* in DQ and JP0d is consistent with previous monitoring of low-temperature Daqu [5,27]. However, its enrichment on vat-associated surfaces indicates that these reservoirs may contribute fungi that become more relevant during later fermentation stages.

In the fungal PCoA, DQ and JP0d occupied nearby positions, whereas TR was separated from them (Figure 4b). HCA further showed that TR formed the most distinct branch, while DQ and JP0d were positioned within the main cluster together with the other environmental samples (Figure 4c). The ordination patterns of the fungal community were similar to those of the bacterial community, with DQ and JP0d forming a tight cluster.

### 3.4. SourceTracker Analysis Identified Daqu as the Dominant Inferred Source of JP0d Microbiota

To visualize the dynamics of dominant microbial taxa after fermentation initiation, the top 10 bacterial and fungal genera from JP0d to JP28d were profiled (Figure S3). In the bacterial community, Lactobacillus became dominant during fermentation and increased toward the middle and late stages. *Weissella* and *Leuconostoc* appeared mainly during early fermentation and then decreased (Figure S3a). In the fungal community, *Saccharomycopsis* and *Issatchenkia* were the dominant genera at JP0d and together accounted for more than 75% of the fungal community. *Issatchenkia* remained relatively stable during fermentation, whereas *Saccharomycopsis* decreased and *Wickerhamomyces* increased in later stages (Figure S3b). These compositional profiles provided a basis for evaluating the similarity between the initial and later-stage microbial communities.

**Figure 5:**
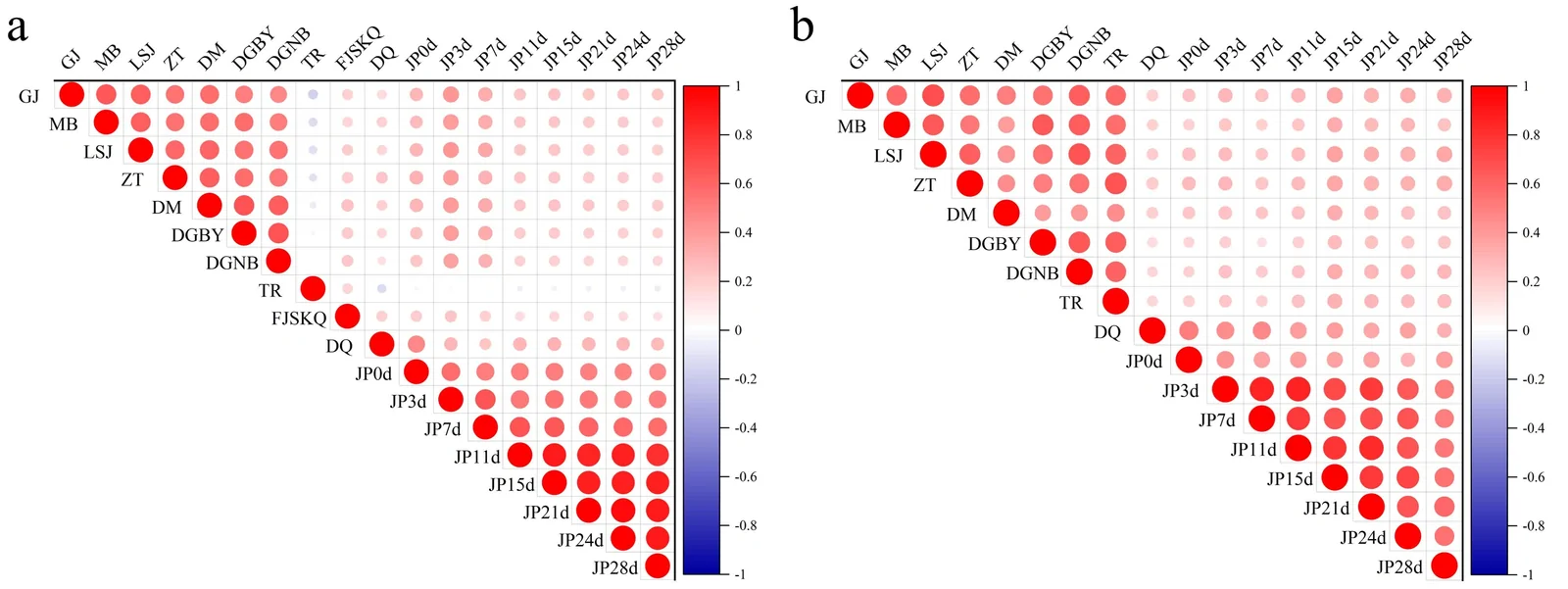
Correlation analysis of microbial communities among Daqu, JP0d, environmental samples, and subsequent fermentation stages. (**a**) Bacterial community correlation analysis. (**b**) Fungal community correlation analysis. Circle color represents the Spearman correlation coefficient; circle size corresponds to correlation magnitude.

To compare JP0d with potential sources and later-stage samples, pairwise correlation heatmaps were constructed using environmental samples and subsequent fermentation stages (JP3d–JP28d) (Figure 5). For bacteria, JP0d was more strongly correlated with DQ (r = 0.47) than with environmental swab samples (r = 0.18–0.26), indicating that the bacterial community of DQ was most similar to that of JP0d. The correlation between DQ and later fermentation stages decreased, reaching r = 0.28 for JP28d. The JP0d–JP28d correlation (r = 0.46) indicated moderate similarity between the initial and final bacterial communities (Figure 5a). For fungi, DQ also showed the highest correlation with JP0d (r = 0.52), whereas environmental samples showed lower correlations (r = 0.18–0.27). The correlation between DQ and JP28d declined to 0.30, and the JP0d-JP28d correlation was 0.39 (Figure 5b). These results indicate that both bacterial and fungal communities in JP0d were most closely related to Daqu, whereas the fungal community showed lower similarity between JP0d and JP28d than the bacterial community.

The bacterial communities of JP0d and JP28d retained moderate compositional similarity (r = 0.46), although *Lactobacillus* became more abundant after fermentation began (Figure S3a). This pattern suggests that the later dominance of *Lactobacillus* developed within an assemblage that still retained part of its initial bacterial composition. Huang et al. (2020) identified *Lactobacillus* as a major member of light-flavor Baijiu fermented grains and reported carbohydrate-metabolism genes associated with this genus [16]. In experiments using isolates from light-flavor Baijiu, Chen et al. (2022) further found that different lactic acid bacterium–yeast combinations produced different ester profiles [28]. These studies provide a functional context for the increase in *Lactobacillus* observed over the fermentation course.

Fungal composition showed a different pattern. The lower similarity between JP0d and JP28d (r = 0.39) coincided with a decrease in *Saccharomycopsis* and an increase in *Wickerhamomyces* (Figure S3b). The relevance of following non-*Saccharomyces* yeasts beyond the initial stage is illustrated by fermentation studies: inoculation with *Saccharomycopsis fibuligera* and *Pichia kudriavzevii* affected yield and flavor in industrial Xiaoqu light-flavor Baijiu [29], while two *Wickerhamomyces anomalus* strains differed in their effects on ester composition in simulated solid-state Baijiu fermentation [30]. The succession observed here therefore identifies changes in the yeast community that merit attention across the full fermentation period.

**Figure 6:**
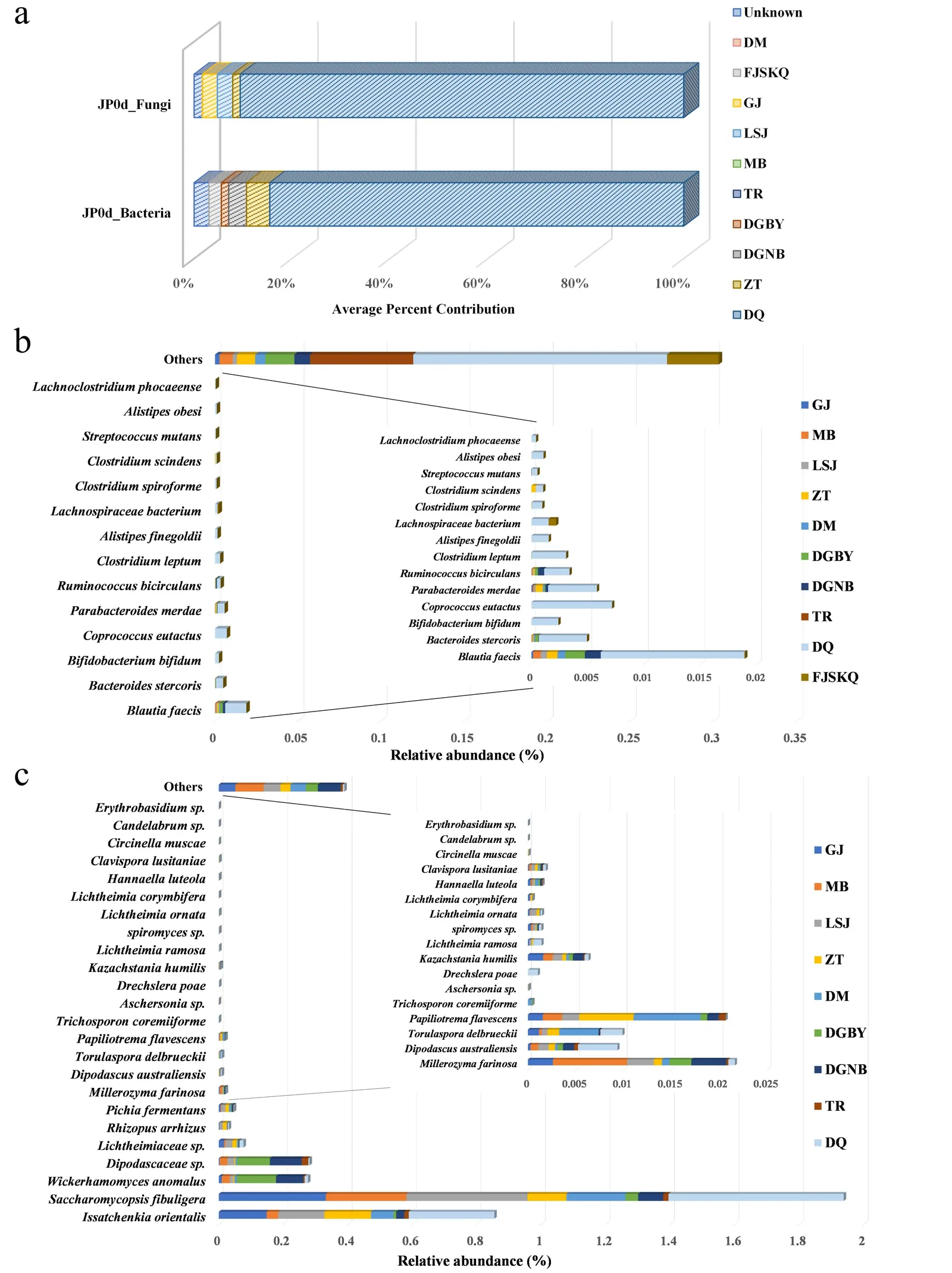
SourceTracker analysis showing inferred source contributions to bacterial and fungal communities in JP0d. (**a**) Average percentage contribution of source communities. (**b**) Source-tracking analysis of bacterial communities in JP0d. (**c**) Source-tracking analysis of fungal communities in JP0d. Unknown means unassigned by SourceTracker conditional on the sampled source set.

SourceTracker analysis further estimated the microbial sources of JP0d (Figure 6). Daqu was inferred to be the dominant microbial source for JP0d, contributing 87.1% of the bacterial and 90.1% of the fungal communities. Environmental sources also contributed, with fermentation-chamber air (FJSKQ) supplying 2.53% of the bacterial community, and equipment surfaces (ZT, DGBY, DGNB) contributing 1.1–5.1%. Fungal inputs from environmental sources were mainly associated with ZT (1.5%), LSJ (3.2%), and GJ (3.0%) (Figure 6a). At the genus level, we further estimated the potential contributions of Daqu and environmental sources to the major genera. For bacteria, several abundant taxa, including *Bifidobacterium*, *Coprococcus*, *Alistipes*, and *Lachnospiraceae*, were almost exclusively derived from Daqu (≥99% potential contribution), while *Blautia*, *Parabacteroides*, and *Ruminococcus* also had Daqu as the major source (66.8–74.4%) with minor environmental inputs. For fungi, *Saccharomycopsis* and *Issatchenkia* were predominantly derived from Daqu, whereas *Rhizopus* and *Lichtheimia* received substantial contributions from GJ, LSJ, and ZT. These genus-level patterns were consistent with the species-level source profiles (Table S1, Figure 6b,c). These results indicate that JP0d microbiota were predominantly Daqu-derived, with smaller but measurable inputs from fermentation-room air and production interfaces.

The source-tracking results explain why the greater diversity of several environmental reservoirs did not translate into a comparable contribution to JP0d. Daqu accounted for most of the inferred bacterial and fungal sources, consistent with its close compositional resemblance to the initial grains. The environmental signal was smaller and varied among air and production interfaces, indicating that these reservoirs contributed to the initial assemblage to different extents. Pang et al. (2018) reported abundant microorganisms on production interfaces and examined their associations with light-flavor Baijiu fermentation [7]. The present source estimates add a quantitative view of the fermentation starting point; JP0d had a predominantly Daqu-associated community with smaller, detectable contributions from the sampled environment.

### 3.5. Co-Occurrence Network and Predicted Functional Potential of the JP0d Microbiome

**Figure 7:**
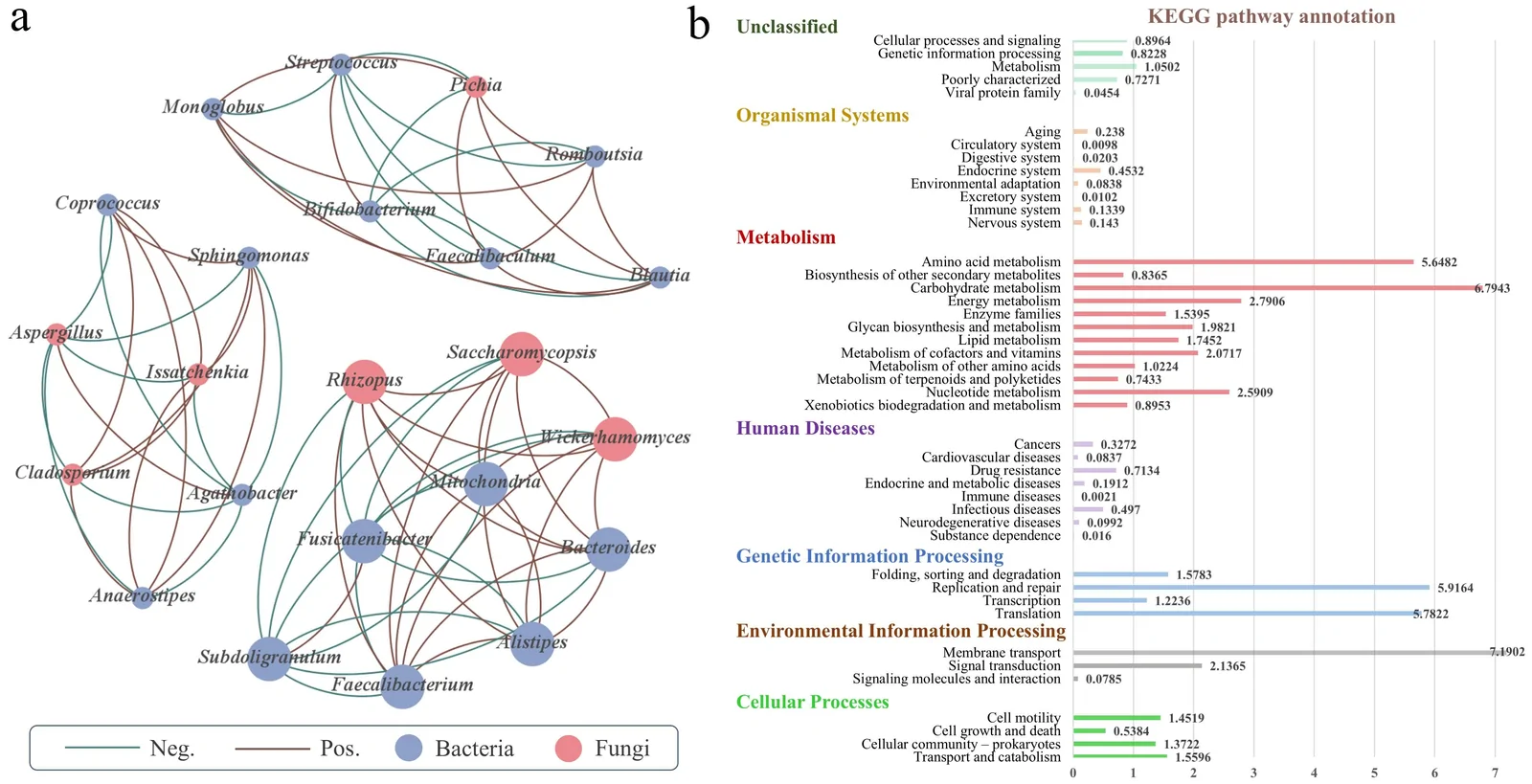
Co-occurrence network and predicted functional potential of the JP0d microbial community. (**a**) Co-occurrence network based on dominant microbial genera. (**b**) KEGG pathway annotation based on PICRUSt2 functional prediction. Neg., negative correlation; Pos., positive correlation. Node size indicates microbial abundance, and line width denotes correlation strength.

To explore potential microbial associations in JP0d, Spearman-based co-occurrence network analysis was performed for dominant bacterial and fungal genera (Figure 7a). The network contained 23 nodes and 78 edges, including 44 positive and 34 negative correlations. Positive correlations accounted for 56.96% of the edges, whereas negative correlations accounted for 43.04%. The average degree, graph density, and average clustering coefficient were 6.783, 0.308, and 0.988, respectively (Table S2). *Saccharomycopsis*, *Wickerhamomyces*, and Rhizopus showed positive pairwise correlations, whereas these fungal genera were negatively correlated with *Fusicatenibacter* and *Subdoligranulum*. These associations indicate potential co-occurrence or exclusion patterns in the initial microbiome.

PICRUSt2-based KEGG prediction indicated that metabolism-related pathways were the most abundant predicted functional category in JP0d (Figure 7b). Amino acid and carbohydrate metabolism were prominent among the predicted metabolic pathways. These predicted pathways may be relevant to the availability and transformation of fermentable sugars and nitrogenous compounds during early Baijiu fermentation. Membrane transport was the most abundant secondary pathway within environmental information processing. Predicted pathways related to translation, replication, and repair were also prominent within genetic information processing. These predicted profiles suggest that the bacterial community in JP0d has the potential for substrate utilization, nutrient transport, and growth-related processes. The prominence of predicted carbohydrate and amino acid metabolism is consistent with the cereal substrate and the metabolic requirements of an establishing fermentation community. Using shotgun metagenomics, Huang et al. (2020) observed an increase in genes related to carbohydrate and amino acid transport and metabolism during the early stages of light-flavor Baijiu fermentation [16], providing a fermentation-stage comparison for the functional profile predicted here at JP0d.

## 4. Conclusions

The present study investigated the microbial sources of initial Jiupei in Fen-flavor Baijiu fermentation. Daqu was identified as the dominant source, contributing 87.1% of the bacterial and 90.1% of the fungal communities, alongside smaller inferred contributions from environmental reservoirs (mainly air and equipment surfaces). These findings advance the understanding of microbial assembly in open solid-state fermentation systems. The results are of practical value to the Baijiu brewing industry for quality control and process consistency, and the traceability framework may also be applicable to other traditional solid-state fermentation industries, such as Huangjiu and vinegar production. Future studies combining isolate-level techno-functional and biosafety characterization with final product chemistry and sensory analysis would help link initial microbial sources to starter potential and final Baijiu quality.

## Data Availability

The original contributions presented in this study are included in the article/Supplementary Material. Further inquiries can be directed to the corresponding authors.

## Supplementary Materials

The following supporting information can be downloaded at: https://www.mdpi.com/article/10.3390/microorganisms14102306/s1, Figure S1. The dilution curves of bacterial and fungal communities. (a) Bacterial communities; (b) Fungal communities. Figure S2. Relative abundance of communities at the phylum level across Daqu, JP0d, and all environmental samples. (a) Bacterial community; (b) Fungal community. Figure S3. Relative abundance of the top 10 microbial genera during the fermentation of Jiupei from day 0 to day 28. (a) Bacterial community; (b) Fungal community. Table S1. Potential contribution of Daqu and environmental sources to the major bacterial and fungal genera in JP0d. Table S2. General information of sequence and alpha diversity in LT-Daqu samples.
