# Supplementary material for: Source Tracking and Functional Prediction of Microbial Communities in Initial Jiupei of Fen-Flavor Baijiu: Daqu-Dominated Sourcing with Measurable Environmental Inputs
Source: Microorganisms. 2026 Oct 10;14(10):2306. doi: 10.3390/microorganisms14102306 (PMC13707915; doi:10.3390/microorganisms14102306)
Supplement: Supplementary file 1 [file microorganisms-14-02306-s001.zip › Supplementary File S1.pdf]

Source Tracking and Functional Prediction of Microbial Communities in Initial Jiupei of Fen-Flavor Baijiu: Daqu-Dominated Sourcing with Measurable Environmental Inputs

Enxiang Zong [1,2], Jiaojiao Zhang [2,3], Baoyue Zhu [1], Baoqing Bai [2,4], Jinhua Zhang [2,4], Ying Zhang [2,4], Yukun Yang [2,4], Sanhong Fan [2,4,*] and Tao Bo [2,4,*]

[1] School of Food and Health, Beijing Technology and Business University, Beijing 100048, China; zongenxiang@st.btbu.edu.cn (E.Z.); 15393116729@163.com (B.Z.)

[2] School of Life Science, Shanxi University, Taiyuan 030006, China; 2024260006@xyafu.edu.cn (J.Z.); baoqingbai@sxu.edu.cn (B.B.); ever840805@sxu.edu.cn (J.Z.); zhangy@sxu.edu.cn (Y.Z.); yangyukun@sxu.edu.cn (Y.Y.)

[3] School of Pharmacy, Xinyang Agricultural and Forestry University, Xinyang 464000, China

[4] Xinghuacun College of Shanxi University (Shanxi Institute of Brewing Technology and Industry), Taiyuan 030006, China

* Correspondence: fsh729@sxu.edu.cn (S.F.); botao@sxu.edu.cn (T.B.)

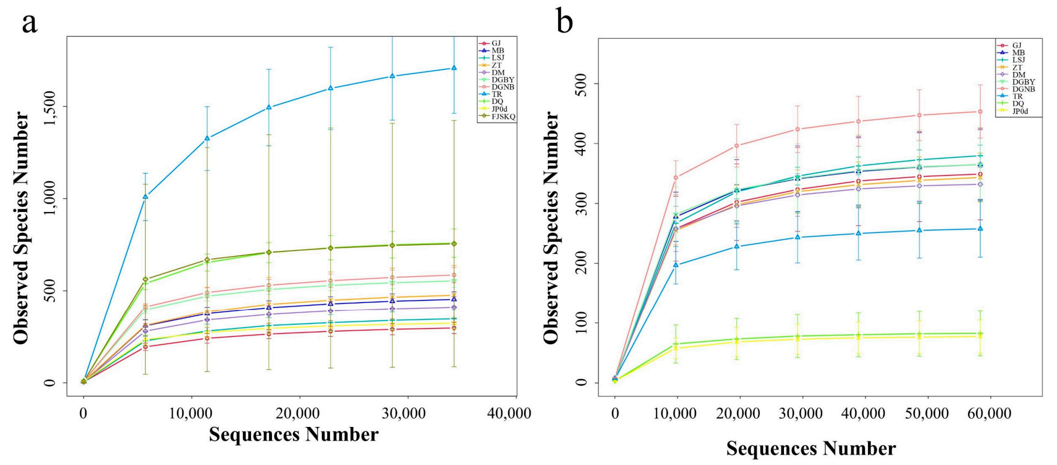

Figure S1 The dilution curves of bacterial and fungal communities. (a) Bacterial communities; (b) Fungal communities.

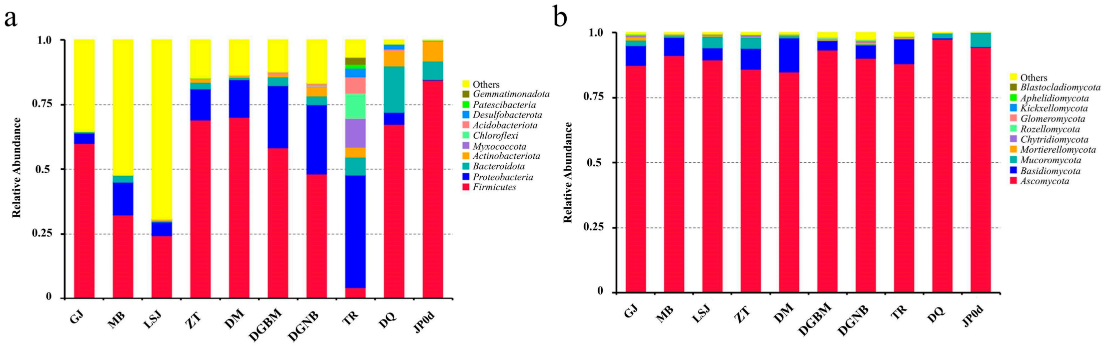

Figure S2. Relative abundance of communities at the phylum level across Daqu, JP0d, and all environmental samples. (a) Bacterial community; (b) Fungal community.

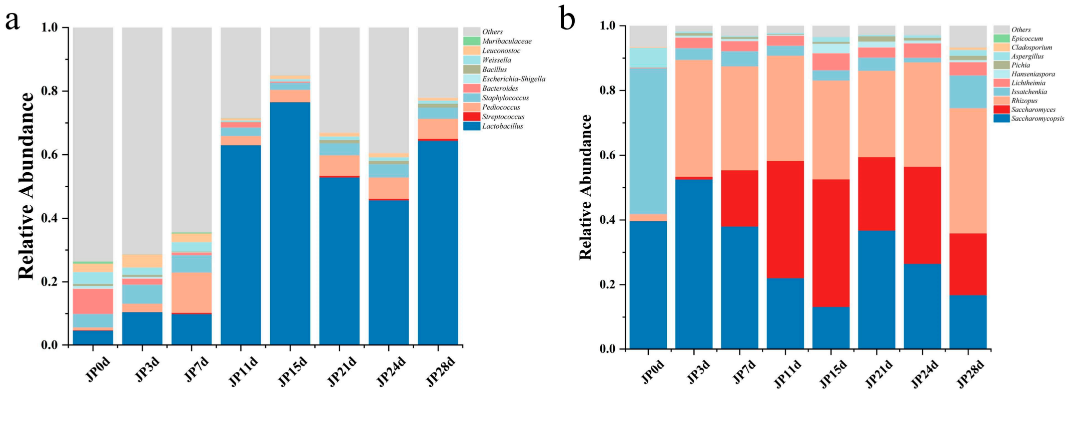

Figure S3. Relative abundance of the top 10 microbial genera during the fermentation of Jiupei from day 0 to day 28. (a) Bacterial community; (b) Fungal community.
